# Full reconstruction of large lobula plate tangential cells in *Drosophila* from a 3D EM dataset

**DOI:** 10.1371/journal.pone.0207828

**Published:** 2018-11-28

**Authors:** Kevin M. Boergens, Christoph Kapfer, Moritz Helmstaedter, Winfried Denk, Alexander Borst

**Affiliations:** 1 Max-Planck-Institute for Brain Research, Frankfurt, Germany; 2 Max-Planck-Institute of Neurobiology, Martinsried, Germany; EPFL, SWITZERLAND

## Abstract

With the advent of neurogenetic methods, the neural basis of behavior is presently being analyzed in more and more detail. This is particularly true for visually driven behavior of *Drosophila melanogaster* where cell-specific driver lines exist that, depending on the combination with appropriate effector genes, allow for targeted recording, silencing and optogenetic stimulation of individual cell-types. Together with detailed connectomic data of large parts of the fly optic lobe, this has recently led to much progress in our understanding of the neural circuits underlying local motion detection. However, how such local information is combined by optic flow sensitive large-field neurons is still incompletely understood. Here, we aim to fill this gap by a dense reconstruction of lobula plate tangential cells of the fly lobula plate. These neurons collect input from many hundreds of local motion-sensing T4/T5 neurons and connect them to descending neurons or central brain areas. We confirm all basic features of HS and VS cells as published previously from light microscopy. In addition, we identified the dorsal and the ventral centrifugal horizontal, dCH and vCH cell, as well as three VSlike cells, including their distinct dendritic and axonal projection area.

## Introduction

In the fly, visual signals are processed in the optic lobe, a brain area comprised of the lamina, medulla, lobula, and lobula plate, each arranged in a columnar, retinotopic fashion (for review, see: [1, 2]). In striking similarity to the vertebrate retina [3], the direction of visual motion is computed within the optic lobe separately in parallel ON and OFF pathways [4–7]. The output of each pathway is represented by a columnar array of neurons called T4 (ON) and T5 (OFF) cells. Within each column, there exist 4 T4 and 4 T5 cells that respond to motion along one of the four cardinal directions and project, according to their preferred direction, into one of the four lobula plate layers [8]. There, T4 and T5 cells provide direct excitatory cholinergic input onto the dendrites of wide-field, motion-sensitive tangential cells as well as onto glutamatergic lobula plate interneurons that inhibit wide-field tangential cells in the adjacent layer [9, 10]. In the fruit fly *Drosophila*, the most prominent lobula plate tangential cells are the cells of the vertical and the horizontal system [11–13] that respond to global optical flow patterns across the field of view [14, 15]. Both groups of cells have also been described in the house fly *Musca* [16] and in the blow fly *Calliphora* [17, 18]. While for *Drosophila*, *Musca and Calliphora*, the number of horizontal cells has been consistently described as 3 across publications, estimations for the number of VS neurons differ. It is believed that *Calliphora* has 10 VS cells, while the most commonly accepted number for *Drosophila* is 6, but contradicting evidence exists as well [13]. In addition, cells similar to VS cells (VS-like or twin VS cells) have been described in the green bottle fly *Phaenicia sericata* [19] and in *Drosophila* as well [13]. For the whole group of dipteran flies, a wide spectrum of tangential cell types and numbers exist across different species [20].

Connectomic analysis of the *Drosophila* brain has yielded important results regarding the wiring of several circuits, including the motion detector in the optical system [21, 22], mushroom body connectivity [23, 24] and evasive behavior circuits in the *Drosophila* larva [25]. All these studies have used 3D electron microscopy to analyze the morphology of cells in the dataset and their connections. This is also an ideal technology to analyze the number and position of cells in a certain area because it reports the cells simultaneously and in an unbiased fashion. Using this technique, a novel cell type (XBC) has been identified in the mammalian retina [26]. In the past, the lobula cell tangential cells in *Drosophila* had been identified in two different ways. One study used the MARCM expression system [27] which relies on the faithfulness of the genetic driver line. Another study [13] used the Golgi staining technique that necessitates for cells to have a characteristic morphology, which makes cell counts dubious. The ideal solution would be a dataset in which to analyze all tangential cells in the lobula plate simultaneously. Here, we present a 3D EM dataset that allows for this.

## Methods

A specimen of *Drosophila melanogaster* wild type was perfused at 4 days old in 2.5% PFA + 2.5% GA in 0.1M Cacodylic buffer. A ROTO staining protocol was employed, using 1h 2% Osmium tetroxide + 3% Potassium Ferrocyanide in 0.1M Cacodylic buffer @ 20°C for 1 hour, then 1% Thiocarbohydrazide @ 54°C (Sigma) for one hour, 2% Osmium tetroxide in H_2_Odd @ 20°C for one hour, 2% Uranyl acetate in H_2_Odd @ 54°C for two hours and then 0.02M Lead aspartate @ 20°C for 12 hours. After dehydration, the sample was embedded in Epon hard (Glaubert) and covered with 200nm of gold.

The sample was then put into a FEI Quanta scanning electron microscope (Thermo Fisher, Waltham, MA) equipped with a custom built SBEM microtome [28]. Cuts were executed so that imaging acquisition proceeded from lateral to medial with a cutting thickness of 25 nm. Initially, images were executed in a 7x11 image grid at 2048 by 1768 pixels, voxel size 11 nm at a sampling rate of 345 kHz. During the progression of the dataset, the field of view and number of tiles was adjusted repeatedly (see S1 Dataset), but never dropped below 7x11 tiles. A total of 10076 slices were cut.

Alignment of the data was executed as described in [29]. Briefly, shifts were calculated by measuring the peak of the cross correlation between all adjacent images and a globally optimal positioning for all tiles was found by applying a least square relaxation to these shift measurements. For the first 7314 slices, the weights of the least square relaxations were adapted to compensate for outliers. The weight was reduced for shift measurements that deviated to far from the expected position or where the maximum of the cross correlation was blurry. For the last 2762 slices, the images were only aligned in z and annotators were asked to find the correspondence between lateral images manually. All reconstructions were done in the skeleton annotation tools KNOSSOS [30] and webKnossos [31]. To annotate start points, processes were identified in the lobula plate that had overly large diameters in lateral cuts throughout the dataset in the layers 92 μm, 99 μm, and 111 μm from the lateral end of the datasets (slices 3664, 3960, 4416). Furthermore, in the layers 153 μm and 183 μm from the lateral end of the dataset the processes in the bundle running medially from the lobula plate were marked (slices 6120, 7312). All start points were created by an expert annotator and then given out to a larger group of annotators. The last 2762 slices were stored in a separate dataset and were reconstructed separately. Therefore all reconstructions have a lateral and a medial part. All skeletons were reconstructed to a redundancy of at least 3-fold, except for the dCH and vCH cell, which were reconstructed only twice and once, respectively. For the volume reconstruction, an annotator went through the annotation and set all nodes to a diameter so that the diameter of the node matched the diameter of the process. Finally, all skeletons were converted by a custom script to hoc files and displayed in Amira (Amira, Thermo Fisher Scientific, Waltham, MA). For the sagittal view only the skeleton parts in the 118 most lateral micrometers of the dataset were shown. All datasets and will be made freely available on https://webknossos.brain.mpg.de upon publication.

For the layer definition, an expert annotator created a skeletonized outline of the lobula plate. With a custom script this was split into an anterior and posterior part and interpolated into two surfaces. These surfaces were considered the definition of the lobula plate and all nodes that overlapped both in the lateral-medial and ventral-dorsal axis with both surfaces were considered for the layer analysis. All skeletons were normalized to a maximum inter node distance of 400nm and projected into this lobula plate model. The lobula plate model was split up into four sublayers of equal width, which were then assumed to be identical to layers 1 to 4. For the boxplots nodes that were further than 50% anterior or posterior to the lobula plate were excluded. Then the boxplot feature included in the MATLAB software was used (MATLAB R2014b, MathWorks, Natick, MA) For the VS cells an explicit consensus skeleton was created for the layer measurement. For cells where no explicit consensus skeleton existed, the average of all skeletons was used as a template for the layer analysis. For the volume measurement, the total volume was assembled from the partial annotations, using the medial part and lateral part (largest component), excluding the soma reconstruction.

For the contact measurement in the axonal tract, the diameter annotations where converted into a voxel representation of the axonal tract (voxel size (140nm)^3^). This calculation was executed for the stretch from 86μm to 203μm from the most lateral end of the dataset. Then, the voxelsation pattern was dilated twice with a cubic kernel of 3x3x3 voxels and eroded twice with the same kernel. Dilation was repeated 7 times, with the restriction that processes can’t grow into other processes. Then, each process was dilated with a cubic kernel of 3x3x3 voxels (while the other processes stayed constant) and the overlap between the dilated process and the others was measured. The number of voxels that overlap was divided by 51 to arrive at the surface area in (μm)^2^. If this measurement showed that two processes did not touch at all, it was verified by manual inspection.

## Results

**Fig 1:**
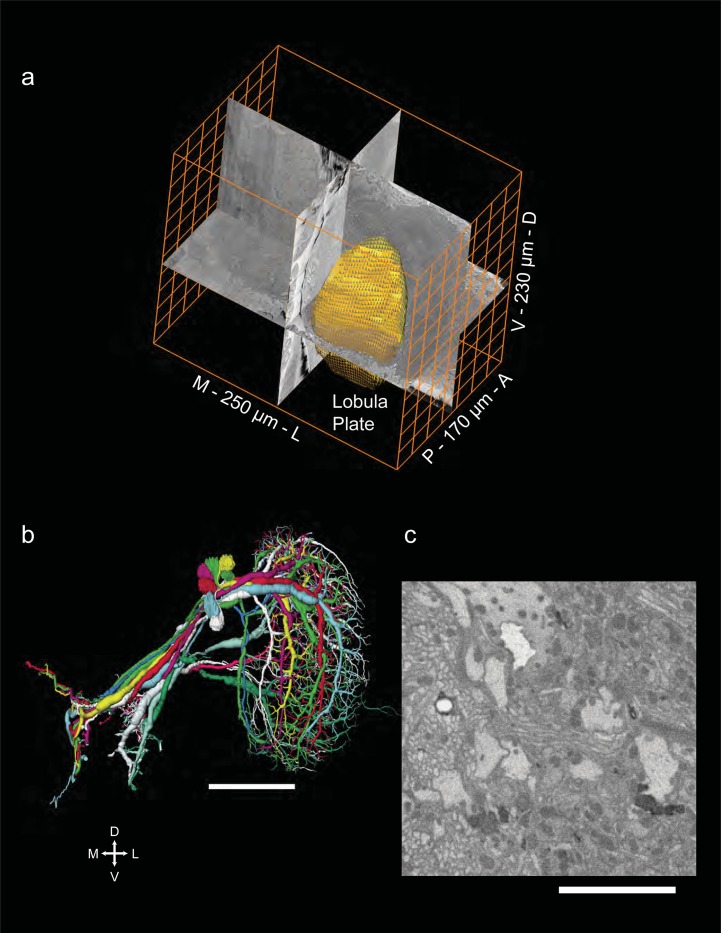
(A) Volume outline of the *Drosophila* lobula plate within the tissue block sized 170 x 230 x 250 μm^3^. (B) 14 reconstructed tangential cells, comprising the three HS cells (HSN, HSE, HSS), two CH cells (dCh, vCH), VS1-6 and 3 VSlike cells. Scale bar 50μm. (C) Example electron micrograph from the lobula plate. Scale bar 5μm.

The lobula plate of the fruit fly *Drosophila melanogaster* has been described as an important center where visual motion information is collected and analyzed by large tangential cells. Here, we map the morphology of a group of lobula plate tangential cells in a three-dimensional electron microscopy data set of the size 170 x 230 x 250 μm^3^ (Fig 1A). To do so, we made annotations starting in 5 different sagittal planes, which were then selectively used for a total reconstructed length of over 50 mm (Fig 1B). The staining and data quality allowed for a complete reconstruction of the cells but not for the identification of synapses (Fig 1C).

### Tangential cells of the horizontal system

**Fig 2:**
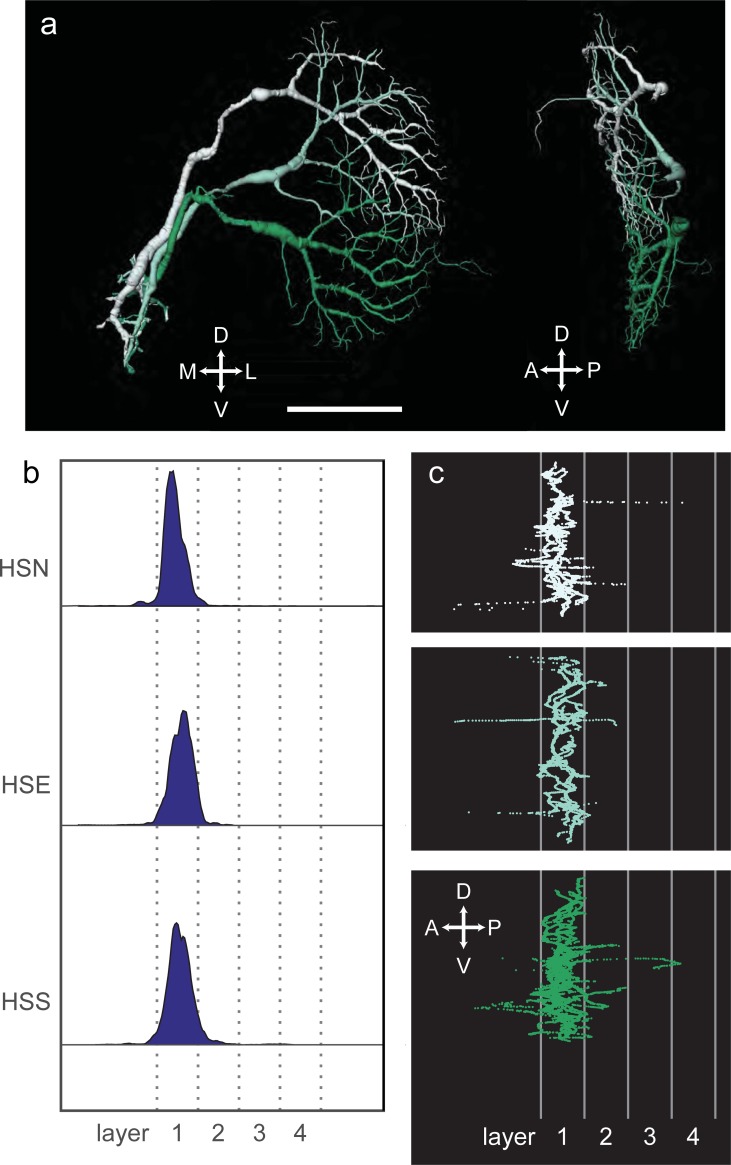
(A) Reconstructions of the three tangential cells of the horizontal system, named HSN, HSE and HSS. Scale bar 50 μm. (B) Histograms of dendrite distribution. (C) Sagittal view of HS cells normalized to layer structure.

We reconstructed the three cells of the horizontal system (Fig 2A). Their dendrites tile, with significant overlap, the lobula plate into a dorsal (northern HS cell, HSN), middle (equatorial HS cell, HSE) and ventral (southern HS cell, HSS) area. Because we have the morphology of the cell co-located with an outline of the lobula plate, we could precisely measure in which layers the dendritic tree resides. These cells restrict their dendritic terminals to the most anterior layer 1 of the lobula plate (Fig 2B and 2C). Their axons join each after leaving the lobula plate. They form a tract separate from the VS cells and terminate in the inferior posterior slope (IPS, [32]), lateral to the VS cells (Fig 1B). Their somata could not be identified as part of the soma cluster of the VS cells. Apart from a few thin dendritic branchlets outside layer 1 of the lobula plate not seen previously, our reconstructions agree in all respects with previous descriptions of these cells at the light microscopic level [12, 15, 33].

**Fig 3:**
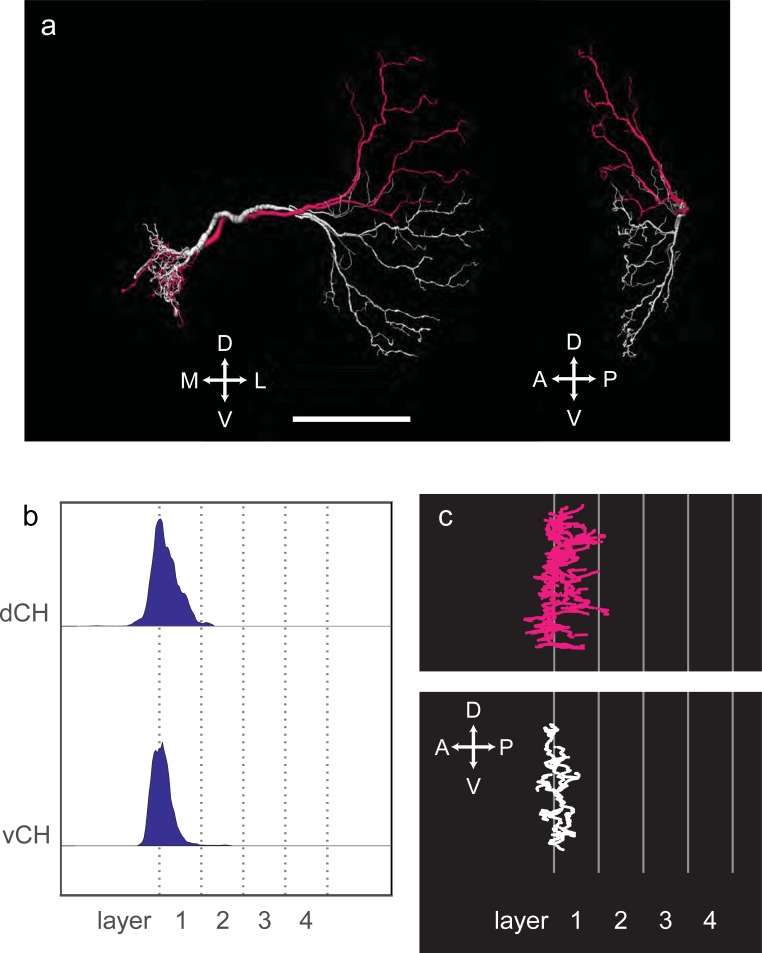
(A) Reconstructions of the two centrifugal horizontal cells, named vCH and dCH. Scale bar 50 μm. (B) Histograms of dendrite distribution. (C) Sagittal view of CH cells normalized to layer structure.

In addition to HS-cells, two other cells of the horizontal system exist in the blow fly, the so-called centrifugal horizontal CH-cells [34]. They tile with significant overlap the dorsal and the ventral part of the lobula plate layer 1 and are called dCH and vCH, respectively. Both of them have an additional dense arborization in the posterior slope of the protocerebrum and a cell body fiber that connects the processes to the soma located on the contralateral hemisphere. We reconstructed two cells from our data set which closely resemble the vCH and the dCH cell in *Calliphora* (Fig 3). As in the blow fly, their arborizations within the lobula plate are restricted to layer 1 (Fig 3B and 3C). Both of them arborize densely in the same volume of the posterior slope, from where a cell body fiber originates. Since our data set is limited to the ipsilateral hemisphere, their cell body fiber could not be traced to their soma. The arborizations within the lobula plate and in the posterior slope are connected through a thicker process. The processes of both cells run intertwined in a common tract.

### Tangential cells of the vertical system

**Fig 4:**
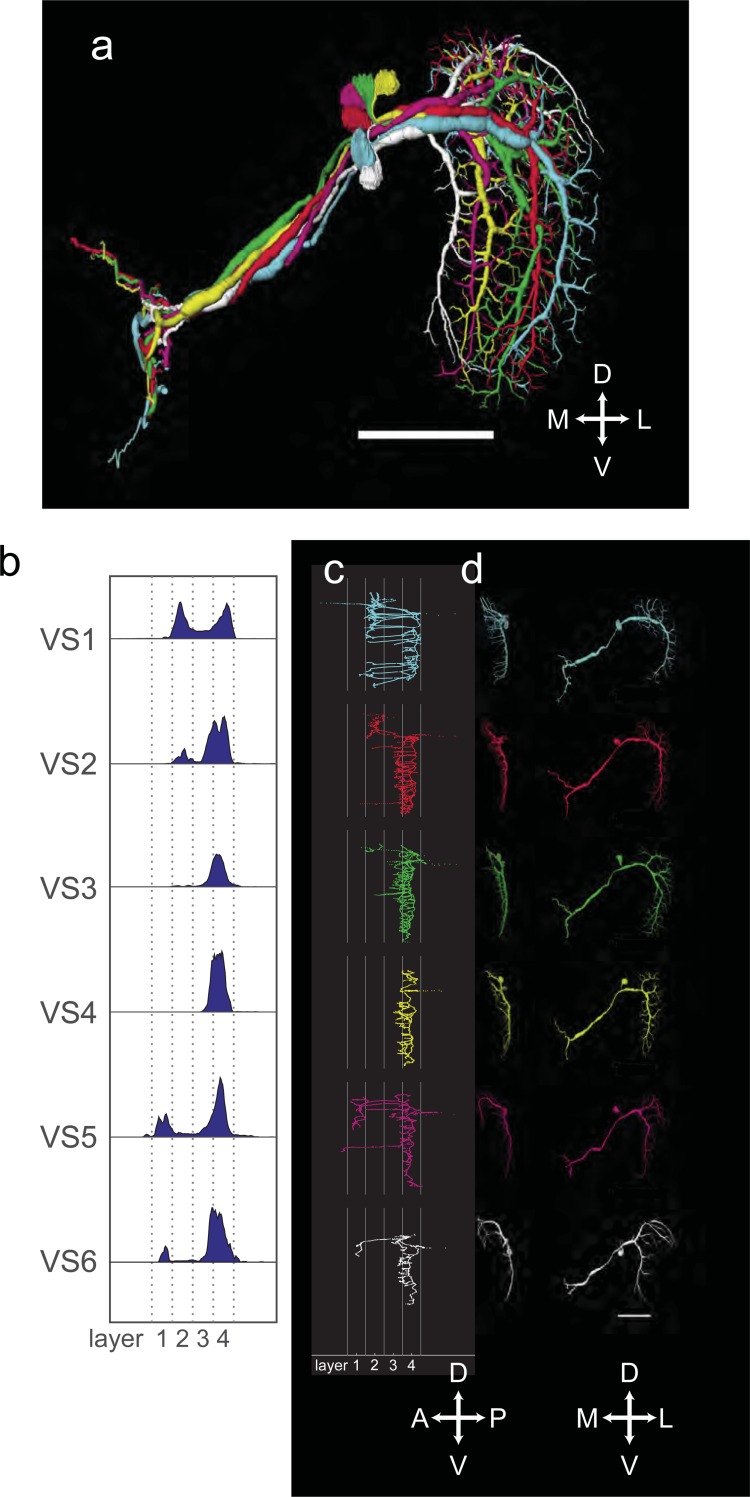
(A) Reconstructions of VS1-6, frontal view. The numbering corresponds to the lateral-medial order of their ventral dendrite. (B) Histograms of dendrite distribution. (C) Sagittal view of VS1-6 normalized to layer structure. (D) Single VS1-6 reconstructions, sagittal (left) and frontal (right) view. All scale bars 50 μm.

The dendrites of the tangential cells of the vertical system (VS-cells) mainly reside in layer 4 of the lobula plate. Here, they cover a rather narrow stripe extending from the dorsal to the ventral edge. 6 VS tangential cells have previously been identified in *Drosophila*. They are named VS1-VS6 according to the position of their most ventral dendrite along the horizontal axis of the lobula plate. We were able to reconstruct all VS cells from our data set (Fig 4).

As described in the literature, VS1 extends many dendritic branches into frontal layers of the lobula plate. Somewhat surprisingly, the anterior branchlets of the VS1 cell extend into layer 2 where information about back-to-front motion arrives [8]. These extensions account for a total of 39% of VS1-cell’s processes within the lobula plate and are found in the dorsal as well as in the ventral part of the dendrite. This might have interesting consequences for the receptive field structure of the cell. Given such extensions in different layers of the lobula plate, the cell’s local motion preferences are expected to vary with the location within the receptive field, as has been reported for VS-cells of the blow fly *Calliphora* [35–38]. Particularly, VS1 of *Calliphora* has been shown to be sensitive to back-to-front motion in the dorsal part of its receptive field [36], as would be expected from dendritic terminals in layer 2 found here for *Drosophila*. The general morphology of VS2 matches what had been previously reported [12], with less complex dendrites than VS1. In addition, however, we found a small patch of innervation into layer 2 in the most dorsal part of the dendritic tree. Again, this should be reflected in the receptive field structure of the cell. We also identified VS3, based on the position of its main dendrite and branching density in the small anterior ventral extension, in agreement with previous reports [12]. Apart from this extension, VS3 confines its dendritic branchlets mostly to layer 4 throughout its dendritic field. Of all the 6 VS cells, VS4 exhibits the narrowest distribution of its dendrites, with 78% of them in layer 4. This mostly agrees with the previously reported results, where only two small branches were described to extend into anterior layers [12]. In case of VS5, its attribution is not as clear as for the other cells. According to [12], a defining characteristic of VS5 is that it has two branches that extend dorsally. This could not be found in our reconstruction. On the other hand, VS5 was described to have a quite heavy tuft that extends anteriorly, which is in agreement with our reconstruction. As the most medial of all VS cells, we identified VS6 in our data set. One defining characteristic of VS6 is that the most lateral part of its dendritic tree is drooping ventrally [12]. This agrees well with our result. A part of the neurite of VS6 extends into the anterior part of the lobula plate, with 9% of dendritic branches reaching into layer 1. Again, as for VS1, this is in agreement with the sensitivity of lateral VS-cells (VS7-10) for front-to-back motion in the dorsal part of their receptive field as described in *Calliphora* [36, 38].

**Fig 5:**
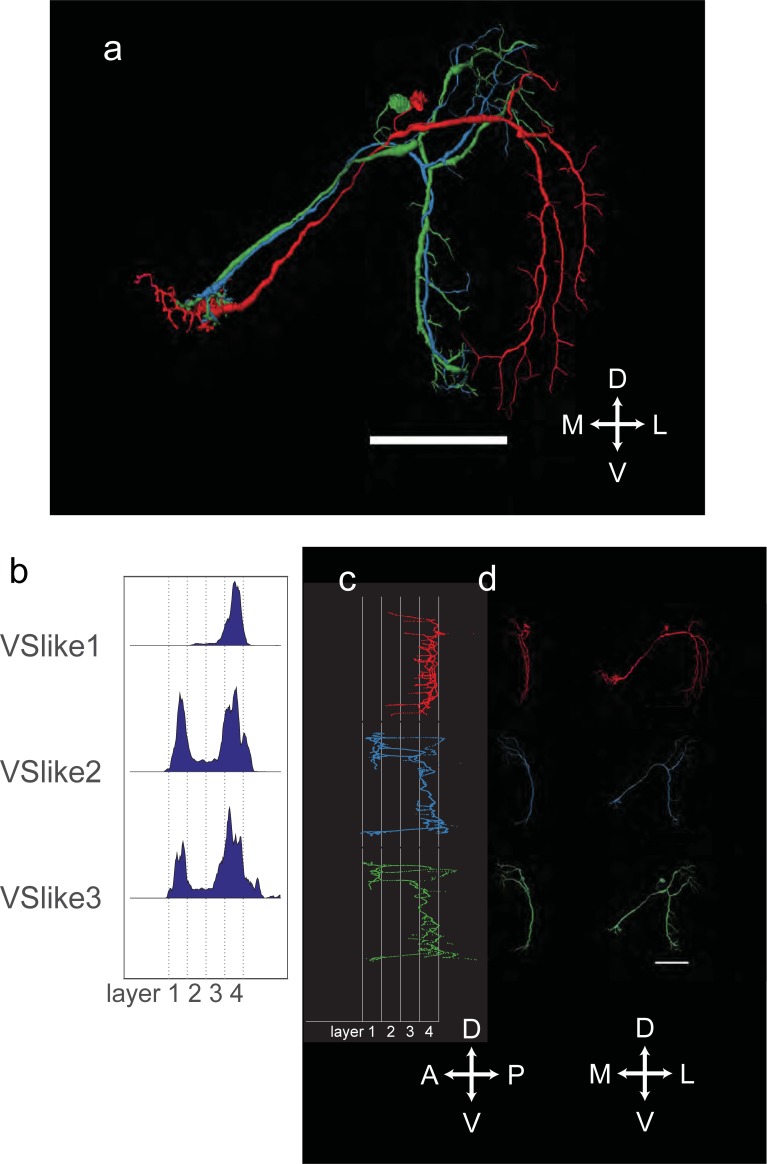
(A) Reconstructions of VSlike1-3, frontal view. (B) Histograms of dendrite distribution. (C) Sagittal view of VSlike1-3 normalized to layer structure. (D) Single VSlike1-3 reconstructions, sagittal (left) and frontal (right) view. All scale bars 50 μm.

In addition to VS1-6, we also identified other cells that resembled the cells of the vertical system (Fig 5A). They differentiated themselves from VS1-6 by the fact that they had smaller volumes. Applying the same scheme as used for VS1-6, we named them VSlike1-3 according to the lateral-medial position of their ventral dendrite along the horizontal axis of the lobula plate. The ventral dendrites of VSlike2 and VSlike3 are closely intertwined; therefore, there is some ambiguity in this labeling. On average, their dendrites are less confined to layer 4 (Fig 5B and 5C). The VSlike cells generally have lower diameters and sparser dendritic branches than VS cells. VSlike1 (Fig 5C and 5D) resembles VS2, with few extensions outside of layer 4. The main difference to the previously described VS2 is the existence of a main branch in the ventral part of the dendrite. VSlike2 and VSlike3 have a rather similar appearance. They both have a strong dorsal extension into the border region of layer 1 and 2. In this sense, they resemble the previously identified VS3 [12]. They differ, however, from VS3 with respect to their sparse dendrites and their small volume. In addition, they have a small ventral extension into layer 1 (Fig 5C and 5D).

### Axonal contacts and terminal regions of vertical cells

**Fig 6:**
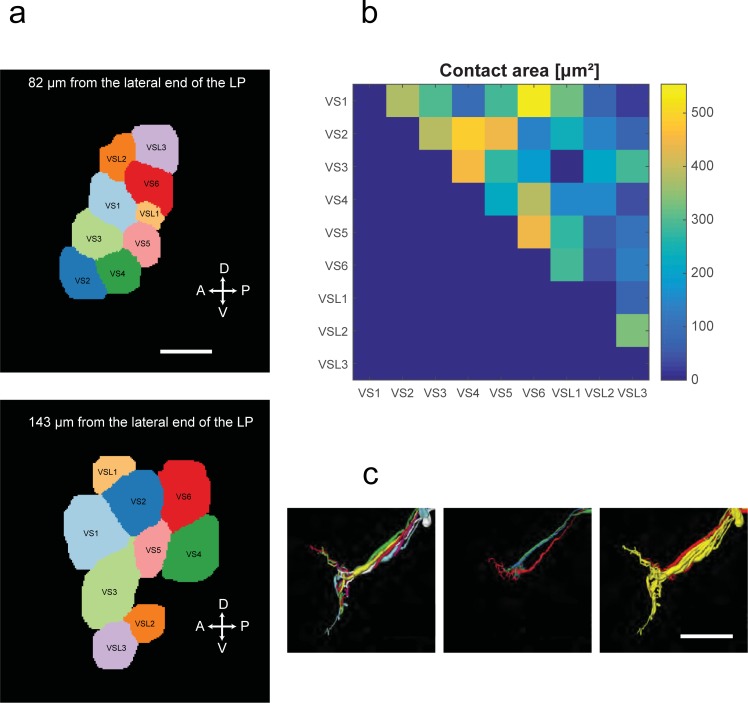
(A) Two example cross sections of the bundle tract. Scale bar 5 μm. (B) Quantified contact area between axons within the tract. (C) Axon terminals of VS1-6 and VSlike1-3 (Left: VS cells only, colored as in Fig 4; Middle: VSlike only, colored as in Fig 5; Right: Both cell types, VS-cells in yellow, VSlike in red). Scale bar 50 μm.

We also measured the relative contact area between the 9 identified vertical cells in the axonal bundle that is running medially from the lobula plate (Fig 6A,B). This measurement is relevant because the absence of contacts between two axons means that they can form no gap junctions. Amongst the VS1-6 cells, all members have contact area with each other (VS1 with VS2, VS2 with VS3 and so on). No contact was found only between VSL1 and VS3 and between VSL1 and VSL2. The contact area between the different members of the VS1-6 cells is consistent with studies regarding gap junction coupling between VS cells in *Calliphora* [33, 39–42] and *Drosophila* [14] as well. The relative position of the axons relative to each other changes frequently, so that overall almost all axon pairs have touched each other (Fig 6A). We closely investigated the axon terminal regions of all VS1-6 cells and the three VSlike cells (Fig 6C). As a common feature, all VS-cells 1–6 have an axon that bifurcates along the dorso-ventral axis and terminates in two regions of the central brain, the superior and the inferior posterior slope (SPS and IPS, [32]), respectively. In contrast, the three VSlike cells only extend their axon into the superior posterior slope, without a ventral branch.

## Discussion

In prior studies using light microscopy, the detailed reconstructions were done in different animals, because datasets with many tangential cells stained simultaneously were too dense for accurate reconstruction of individual cells. This limitation does not exist anymore for multi-color labeling (‘Brainbow’: [43]) and in particular 3D EM datasets, which we used to sort and analyze all cells in one specimen., The objective of this study was to detect lobula plate tangential cells and their relative position in an unbiased fashion that required neither genetic driver lines nor random subsampling. With respect to the three HS cells and the six VS cells, we found an overall agreement with the study of [12]. However, regarding the fine detail of dendritic ramifications in VS cells, there are some discrepancies as well. Whether these differences can be accounted for by the different methods or whether they represent inter-individual variability remains to be determined by future experiments. In addition, we reconstructed three VSlike cells that could be homologues to the twinVS cells (*Phenicia sericata*: [19]; *Drosophila melanogaster*: [13]) as well as the dorsal and ventral centrifugal horizontal dCH and vCH cell. Applying a redundant procedure to generate start points (skeletons from same cells were traced up to 12 times), we are optimistic that we found all VS candidate cells in the lobula plate with a volume larger than 500 μm^3^. Due to ambiguous tracing, other tangential cells as shown in [44] or [45] could only incompletely be reconstructed from our data set.

As revealed in an ultrastructural study, CH cells in *Calliphora* are both pre and postsynaptic within their lobula plate arborization, and purely postsynaptic in their protocerebral arbor [46]. In addition, CH cells form electrical dendro-dendritic synapses with HS cells [47]. This particular wiring scheme leads to a spatial blur of the motion image on the CH cell dendrite, and, after inhibiting small-field selective neurons [48], to an enhancement of motion contrast [49]. The existence of CH cells in *Drosophila* makes it plausible that they fulfill a task similar to what has been proposed for *Calliphora*, but whether this is true remains to be seen by future experiments.

Investigating the contact area in the axon bundle and the terminal region, we found that almost all VS cells have contacts in the axonal bundle. This offers the possibility that VS1-6 cells form gap junctions between themselves, as has been found by dual electrical recording from pairs of VS-cells in *Calliphora* [33, 39, 41, 42] and by dye-coupling in *Drosophila* [14]. Together with the motion preferences of their presynaptic, motion-sensitive T4/T5 neurons, such electrical synapses can strongly influence the size and local properties of the receptive field of tangential cells [41].

We found that VS1-6 cells and VSlike cells terminate in different, partially overlapping regions within the protocerebrum. This hints to differences between these cell groups with respect to their post-synaptic partners. In the blow fly *Calliphora*, VS cells are connected to a neck motor neurons as well as to a group of descending neurons called ‘DNOVS’ cells [50–53]. The recent generation of more than 100 different driver lines for individual descending neurons in *Drosophila* [54] offers a great opportunity to study the connectivity of VS and VSlike cells to these and other neurons.

## Supporting information

S1 Dataset Complete set of reconstructions and accompanying dataset information. (ZIP)
