# Supplementary figures and images for: Full reconstruction of large lobula plate tangential cells in Drosophila from a 3D EM dataset
Source: PLoS One. 2018 Nov 28;13(11):e0207828. doi: 10.1371/journal.pone.0207828 (PMC6261601; doi:10.1371/journal.pone.0207828)

Supplement: S1 Dataset — (ZIP) [file pone.0207828.s001.zip › Diameter hoc/empty.tif]

Supplement: S1 Dataset — (ZIP) [file pone.0207828.s001.zip › Diameter hoc/emptywhite.tif]
